# Barriers and facilitators to implementation of a community wellbeing service for black, Asian and minoritised young people

**DOI:** 10.3310/nihropenres.13912.2

**Published:** 2025-09-23

**Authors:** Sharea Ijaz, Shumona Salam, Jo Williams, Geraldine Smyth, Deborah M Caldwell, Katrina Turner

**Affiliations:** 1 University of Bristol Bristol Population Health Science Institute, Bristol, England, UK; 2 Bristol City Council, Bristol, England, UK

**Keywords:** Adolescent, Mental Health, Ethnicity, Community setting, Qualitative Research, Complex health interventions

## Abstract

**Background:**

Young people from minoritised ethnic backgrounds experience greater mental health needs and face greater challenges when accessing mental health support. We evaluated implementation of a new mental wellbeing service for minoritised young people in an urban youth centre.

**Methods:**

We evaluated the service during its first 12 months of implementation. We held twelve interviews with four service practitioners and three paired interviews with six young people. Fieldnotes were taken and used to contextualise interview data. Practitioners recorded young people’s attendance. Qualitative data were analysed thematically. Attendance data were analysed descriptively.

**Results:**

The service included Four components: a weekly two-hour session with mental health practitioners in the youth centre, opportunistic wellbeing conversations and activities, mentoring, and referrals to therapeutic support. It was developed iteratively to allow time for relationships between practitioners and with young people to develop and for intervention to be tailored to the setting.

Implementation was facilitated by the setting’s positive influence, practitioners’ lived experience, iterative development of the service, and establishing trusting relationships. Barriers included the informal nature of activities, slow service implementation, and young people’s inconsistent attendance and reluctance to engage with the service. 94 young people attended at least one session.

**Conclusion:**

Successful implementation of wellbeing services in community settings for minoritised young people can be affected by the informal and relaxed nature of the setting and the activities delivered, and the extent to which young people are willing to engage. Additionally, it requires relationship building and flexibility in delivery and pace. Future development and evaluation of similar services should consider these requirements.

## Plain Language Summary

Young people from ethnic minority backgrounds have greater need for mental health support. Yet they often face difficulties in finding this support. We evaluated a new mental health support service for these young people in a youth centre.

Over 12 months, we interviewed service practitioners and young people, took fieldnotes, and recorded young people's attendance. We analysed all this information to identify what helped implementing such a service and what didn't.

The new service had four components: weekly sessions with mental health practitioners, wellbeing conversations and activities, mentoring, and referrals to other services. It evolved over time as relationships were built and was tailored to the environment in the youth centre.

What helped the implementation of the service was the supportive environment, providers' lived experience, service flexibility, and establishment of trust. Whereas slow service delivery, unstructured activities, irregular attendance of young people, and their reluctance to engage hindered the implementation.

Relationship building and flexibility of a service are required for successful implementation of mental health support services for young people from minoritized ethnicities. Future service development should consider these factors.

## Introduction

Children and young people (CYP) from Black, Asian, or minoritised ethnic backgrounds are underrepresented in NHS services despite higher rates of mental health conditions ^1– 4^. Reasons include CYP facing greater challenges in accessing mental health support ^5^, not knowing about services or locations, language and culture differences between themselves and health professionals, and higher mental health stigma in minoritised communities ^1, 5– 8^. Minoritised CYP are more likely to be referred to Child and Adolescent Mental Health Services through education, social services, or judicial pathways rather than the usual primary care route ^9, 10^. They often seek support from informal services, community organisations, or family and friends ^1, 9– 12^. 

Providing culturally appropriate mental health services could address these barriers ^13– 16^. The UK mental health framework encourages the use of community assets, such as youth groups to support CYP wellbeing ^17^. Community organisations can provide context-specific preventive mental health services, but evidence indicates gaps in evaluation of these services ^18– 20^. A new community-based mental wellbeing service was commissioned by Bristol’s local authority to improve wellbeing support and access to mental health services for CYP from Black and Asian minoritised ethnicities. To set up the service, the local authority organised a meeting with Black and Asian led CYP support organisations in the city to identify those willing to collaborate on service delivery in a community setting. The organisations in one locality who agreed were contracted to collaborate for developing and providing the service and supporting an evaluation. The key service aims were to support minoritised young people’s mental wellbeing and facilitate referrals to mental health services, where appropriate. The service was set in a youth club and intended to be both universal (through games and activities for all attendees) and targeted (through mentoring and/ or referral for at risk CYP). We evaluated this service in its first year to identify barriers and facilitators to implementation. This paper presents findings from the evaluation and highlights what should be considered when developing and evaluating similar future interventions.

## Methods

### Participants

Eligible study participants were the four practitioners delivering the service and all the minoritised CYP aged 11–17 registered at the centre attending the service. We aimed to purposefully sample CYP of varying age and gender for interviews. A £10 shopping voucher was given to CYP who participated in interviews. 94 CYP attended at least one wellbeing session during the course of the study. All of them were shown study flyers and leaflets and approached for participation in the study. Six CYP and all four service providers from the centre were interviewed. Their characteristics are not described to avoid identification.

The providers were a youth worker and a manager, and two mental health practitioners. They visited the centre weekly to deliver the service. All four were interviewed on three occasions between December 2022 and March 2024; at the start of service implementation, then after seven, and 12 months. Interviews lasted one hour on average (range 37–74 min). Five interviews were conducted face-to-face, at the youth centre or at the mental health organisation’s office, and seven were conducted by phone.

CYP were reluctant to participate in the evaluation and only three paired interviews were conducted. The six young people interviewed were between 11 and 15 years (Median age 13 years), included both boys and girls and represented a range of minoritised ethnic backgrounds. These interviews lasted, on average, 30 minutes and were held in a private room at the centre between May and November 2023.

### Procedure and measures

The new service was set in a community youth centre located in a deprived urban area with a diverse population (30–35% ethnic minority) ^21^. The ethnic makeup of the youth centre attendees was varied, including CYP with Black, Asian and mixed heritages in majority.

The service hub was a 2-hour, weekly, youth activity session run for 11- to 17-year-old CYP in the youth centre where mental health practitioners were present. The aims of the new service were broad, so the specific design and professional activities of the service were not decided a priori and were developed as service was implemented. It was co-developed and facilitated by two youth practitioners from the community centre and two specialist mental health practitioners from the mental health organisation (the service ‘providers’). Majority of the youth and mental health practitioners were from the same community and of Black Asian or Mixed ethnicity. The practitioners discussed and agreed on what the service would comprise of and developed it together, based on their knowledge and experience of looking after minoritised CYP and their own lived experience of being from minoritised communities. They therefore understood the reluctance and sensitivity around mental health. 

We held in person and online conversations prior to and during the study with parents and guardians of children attending the youth centre to inform them about the study and enhance the reach. These conversations indicated that parents trusted the setting providers for looking after their children because of their longstanding presence in the community and shared values.

One author (SS), a senior research associate experienced in qualitative methods and with a master’s in public health, visited the centre and met with practitioners prior to evaluation starting. She was from an ethnic minority background, as suggested by our Public and Patient Involvement work (see below in section titled Public and Patient Involvement). Participants were told that SS was the researcher employed on the study to conduct the qualitative work. She attended all the sessions where all providers (two centre staff and two mental health practitioners) were present, and when the centre was open to CYP aged 11 to 17. During these visits, SS took fieldnotes based on what she observed, such as physical description of the community centre and the activities carried out. These notes provided contextual data for the providers’ and young people’s accounts.

 Throughout the evaluation, SS invited providers and the CYP attending the activity session to interviews, to explore their views and experiences of the service. Individuals approached for interview were given an information sheet that described the aim of the study and what taking part would entail. Individuals could read this sheet at their leisure and ask questions about the study. SS secured written informed consent prior to interview and conducted all the interviews. When the participant was under 16 years of age, consent was obtained from the guardians or parents of the CYP, as well as the CYP themselves.

For interviews with service providers, a topic guide was used to ensure consistency across the interviews. The same guide was used at each of the three time points. It was based on the purpose of the interviews and informed by relevant literature, knowledge of the planned intervention and discussions within the research team. It included open-ended questions to elicit details and description from the participants about the service, its implementation, and the barriers and facilitators to service delivery. The interviews were held at a private location in the centre or via phone, at a time convenient to the participant.

For interviews with young people, a flexible and open-ended topic guide was designed to explore their awareness and experiences with the service. This guide was also refined in conjunction with our young people’s research advisory group (see under Patient and Public Involvement). The topic guide included open questions about their perception of mental health and of the new service. A guide was also developed for the interviews with CYP who were referred for mentoring or additional mental health support. It included questions about young person’s perceptions of the additional support and referral components, and processes and how it could be improved in future. (see Supplementary data for all topic guides ^22^).

### Data management and analysis

The interviews were audio-recorded and fully transcribed professionally. All audio recordings were deleted within three months of interview. The transcripts will be kept securely for 10 years. All information is stored safely and securely by the University of Bristol – in line with the General Data Protection Regulations and the University of Bristol’s Data Protection policy.

In terms of analysing the interview data, following the principles of grounded theory ^23^, data collection and analysis proceeded in parallel, so that findings from earlier interviews informed the focus of later data collection. The interview transcripts were analysed thematically, using the approach suggested Braun and Clarke ^24^. Data collection and analysis proceeded in parallel, so that findings from earlier interviews informed the focus of later data collection. This entailed a subsample of all transcripts being independently read and manually coded by SS and KT, who then met to discuss their coding and interpretation of the data, and to develop a coding frame which reflected themes identified in the data. Once the coding frame had been agreed, transcripts were uploaded into NVivo v14 ^25^ and electronically coded. As data collection progressed, new codes were added to the coding frame and transcripts that had previously been coded were re-coded where necessary. Finally, codes were grouped together to develop categories and collated into potential themes and sub-categories. This inductive approach to the analysis ensured findings were based on participants’ accounts and not coded according to the researchers’ pre-existing assumptions or priorities. Researcher bias was also controlled for through the double coding of transcripts and regular discussion between SS and KT about how findings related to the data set. In addition, during data collection, SS discussed interim findings with providers to check our interpretation of the data. Whilst it was felt SS’s ethnicity had facilitated access to the centre and the interviews with providers, it was also felt her gender had hindered the extent to which young men, particularly the 16- and 17-year-olds, using the centre were willing to take part in the research.

In terms of analysing the quantitative data collected by the providers on CYP’s attendance at wellbeing sessions, and referrals to other services over the 12 months, these data were analysed descriptively using Excel.

### Ethical approval

Ethical approval was granted by the University of Bristol Faculty of Health Sciences Research Ethics Committee on 30 ^th^ Nov 2022 (application ID13984).

### Public and Patient Involvement

We worked with the service providers and an independent young people’s research advisory group (YPAG) prior to evaluation and throughout the study. They helped us in developing participant information materials, choosing outcomes and interpreting the findings. A YPAG is a group of volunteer young people who advise on research concerning young people using their lived experience and age specific views. Members of the YPAG consulted were all from minoritised ethnic backgrounds.

## Results

Fifty service sessions were held over 12 months and 94 of the CYP registered at the centre attended at least one of these sessions. The proportion of sessions attended ranged from 2% to 58% and only 19 CYP attended >=20% of the sessions. Five of the 94 CYP were offered 1:1 additional therapeutic support off-site. None had taken up the offer, during the evaluation period.

We start by describing the centre and then detail the service content and implementation. Finally, we present facilitators and barriers to implementation, and the service impact. Quotes are used to illustrate findings and have been tagged with a participant’s unique identifier number indicating whether a provider or CYP is being quoted.

### The centre

Providers explained the centre had been offering community-based services for 40 years, serving multiple generations of local families, primarily from minoritised ethnic backgrounds. The centre already offered a variety of skill-building activities, such as sports, music production, games, movies, cooking, arts, and crafts. Activities were overseen by youth practitioners, most of whom were from minoritised ethnic backgrounds. The CYP interviewed recalled engaging in activities, and described them as soothing and as building their skills and confidence.

Participation in activities was not mandatory and CYP could move freely within the space, using it predominantly to socialise with their peers and friends. CYP described how this meant the centre provided them with a space to relax and feel safe:

*“Maybe like how to cook for yourself and how to speak to people and just talk to people, anyone, and that it would be safe …safe space ...” (CYP02, paired interview)*
*“… it doesn't feel like at school when it's like you don't really have a choice. I'm more relaxed here… the staff just let you know every time and then you can just choose what you want to do…” (CYP01, paired interview*)

### Service content

The providers stated that the aim of the service was to ensure a referral pathway for CYP to access mental health services and to normalise mental health and wellbeing conversations among centre attendees. Providers explained the service was being developed iteratively, on-site, over several months, and included four components:

(i) Weekly presence of mental health practitioners at the Centre.
(ii) Identifying CYP in need of further support and referring them to appropriate services.
(iii) Opportunistically embedding discussions and activities about mental health into conversations and centre activities
(iv) Offering drop-in sessions for one-to-one or group mentoring.

### Service implementation

Service implementation was slow. Providers explained this was because it was a novel offering and that they had limited experience of such service implementation. They noted additional time was needed to build mutual understanding and trust between the youth workers and mental health practitioners, who had not previously worked together. They also felt the service needed to be developed naturally, through trial and error, so that it fitted within the constraints of the setting and was tailored to the CYP’s needs. 

### Facilitators to service implementation

Facilitators to implementation included building trust and relationships with service users and between organisations, and tailoring activities to context.

***Fostering trust and building relationships***. Providers stated building trust and relationships was crucial to successful service implementation. They mentioned that their expertise and lived experience helped them recognise the stigma and apprehension surrounding mental health in minoritised communities, and be responsive to the needs of these communities:

*“We’re known as being a project that not only provides a service but connects with young people that we work with on a deeper level than most practitioners can because we understand the lived experience because we all are practitioners from the lived experience....”* (SP3 Interview)

Providers explained their approach transcended the traditional therapist-client dynamic, aiming to cultivate informal, youth-friendly relationships akin to youth workers, to mitigate power imbalances and cultivate meaningful connections with the CYP:

*“…I’m very much conversing and engaging with them in vernacular with a presentation that very much matches them. With the core principles of building slow trust…it’s not going in and rushing an engagement or rushing an interaction or connection.”* (SP3 Interview)

Initially, the mental health practitioners did not introduce themselves as such to the CYP, in case this label discouraged communication:

*“… I didn’t advertise that fact (that they were a mental health practitioner) because I don’t think, when you’re developing relationships, you want to be watched…going forward…I’d probably want to use language that would be more understood with children, to explain to them better because some don’t even know what mental health is, or have heard of it as a buzzword on TikTok. So, at the moment, I just like to be the friendly face that they can come to…”* (SP1 Interview)

Additionally, mental health practitioners mentioned their positive relationship with centre staff facilitated the process of them being accepted by the CYP.

***Trust building between the organisations***. Ongoing engagement, knowledge-sharing, and training (of youth centre staff by the practitioners from mental health organisation) facilitated the development of a collaborative relationship between the two organisations. This fostered mutual understanding and trust, enabling staff at the centre to seek guidance in addressing specific challenges related to safeguarding, engaging disengaged young people, and responding to young people’s mental health needs.

***Tailoring activities to the context.*** Providers explained that centre activities were determined by CYP interests and participation was optional. This meant that while the providers wanted to implement structured educational activities, uptake depended on the CYP’s willingness to engage.

*” I think the approach here is slightly different where it’s a bit more run by the children and things are quite relaxed and unstructured, which is its complete beauty, but does have difficulties when trying to implement a more structured…”* (SP1 Interview)

For this reason, providers opportunistically incorporated mental health conversations and activities into their usual interactions with CYP. Examples included regularly checking in with CYP about how they were feeling and encouraging them to discuss something positive or challenging that happened during their day. This was also reflected in the CYP interviews. Although CYP could not recall any specific mental health activities, they mentioned providers checking-in on them, having conversations about their emotions, and discussing positive and challenging events. It was also evident that they felt the providers genuinely cared.

*“…they genuinely want to know how I feel, not just for the sake of asking.”* (CYP 03 paired interview)

### Barriers to service implementation

Barriers to implementation mentioned by providers included inconsistent attendance of CYP, the unstructured nature of activities delivered in the centre, slow intervention delivery, and the CYP’s reluctance to engage in wellbeing services or conversations.

***Inconsistent attendance***. CYP’s inconsistent attendance hindered the development of trust and rapport with the providers, and also affected the continuity of service delivery.

*“…Young people’s attendance is quite inconsistent. So will show up for a week, two weeks. We’ll build a bond. They’ll disappear for a week. So, consistency is also a barrier that we’re facing, which is a fundamental pillar to our work as practitioners.”* (SP3 interview)

***Informal and relaxed structure***. Providers repeatedly highlighted a ‘tension’ between the formality of the service and the informality of the community setting. To engage effectively with CYP, providers had to navigate around the habitual actions and expectations of CYP attending the activities:

*“…the young people…have particular expectations of the space…So people are acting habitually in the space. They will go to the studio. They will hang out, stay on their phones, chat with each other, and disengage from people and professionals in the space because that’s their space and that’s their time. Working alongside established habitual actions within the space, and what young people themselves expect from that space…that’s also been a bit of a barrier for us in terms of being able to work our angle in the space.”* (SP3 Interview)

***Slow pace of service delivery***. All providers acknowledged that service implementation was slower than anticipated. This was partly due to the service being developed through trial and error. Implementation was also slow because the centre needed to develop the safety procedures required when providing a mental health service. It took 12 months for all four service components to become operational. This delay contributed to providers being unsure of their roles within the service. As all the CYP we interviewed were not aware of the new service, this operational delay likely contributed to a lack of service visibility to CYP.

***Hesitancy in engaging with service and providers.*** Providers mentioned that CYP did not want to discuss mental health or access further support when it was offered. They suggested that, in the early phases of the service, this might have been because they were not familiar with the mental health practitioners. They also commented that it could be due to the perception that participating could signal that CYP had problems, underscoring the need for practitioners to approach them with care and sensitivity.

*“……You’ve got to be more therapeutic with your approach or more mindful with your approach because we don’t want them to feel like oh, there’s something wrong with me why this mental health person keeps trying to talk to me…”* (SP2 Interview)

CYP accounts suggested that their reluctance to engage could also be because they were unsure what was meant by 'mental wellbeing' or 'emotions', and what service and activities were being offered. They also mentioned not seeing the service as relevant to them, as they considered their mental health was fine. CYP were also unsure how to discuss their mental health and explain how they felt.

“ *I think it [activity with SP03] helped us understand our emotions a bit more because sometimes you just don't understand what you're feeling and like you just… Well, you can't understand it. …. Yeah. I think it helped me understand emotions because when you describe them [pause] like it helps you understand them because like people just say emotions, but they don't really understand what that actually means.” (CYP02, paired interview)*

## Impact of implementation

During follow-up interviews, providers emphasised they had established relationships with CYP and between the two organisations, despite slow implementation. They had started offering mentoring sessions and had developed a clear referral pathway for CYP needing specialist mental health support. Although they recognised the process of trust building had been slow, it had led to CYP opening up and confiding in the providers:

*"One of the young girls became particularly close with our community mental health practitioner, and she’s been able to confide in things that have been upsetting her."* (SP2 Interview)

Providers noted that once a collaborative relationship had been established between the two organisations, they were able to respond very quickly to challenges. This was demonstrated by the provision of additional drop-in sessions and support for CYP affected by a violent incident that occurred in the local community, during the study period.

*“……[we were] able to layer on another support… because we have this programme in place already. That was a very big benefit the fact that we have got this sort of collaboration…it’s allowed us to be more readily responsive…”* (SP2 Interview).

## Discussion

Successful implementation of a community-based wellbeing service for minoritised CYP was facilitated by the positive regard of the setting, lived experience of service providers, iterative and tailored development of the service, and trust building between all those involved. Barriers included the informal or unstructured nature of centre activities, slow service implementation, CYP’s inconsistence attendance and reluctance to engage.

Services implemented in community centres can positively impact CYP’s wellbeing, particularly for those from marginalised backgrounds ^26^. Our findings describing building trust and relationships, following CYP’s preferences, and collaborative development, have been identified previously as facilitators to implementation of interventions for CYP’s mental health ^27^. 

The profile of CYP attending community youth settings has shifted since the 1980s, from mostly marginalised backgrounds to now mostly children from well-off and safe neighbourhoods ^26^. This may be due to long-standing funding limitations and the current cost of living crisis. Recognising these trends, this service, made possible through local authority funding and community organisations, aimed to embed local provision for minoritised CYP.

Our study shows that preventative services for marginalised CYP are feasible in community settings if time is available for trust building and tailoring to children’s preferences. In-depth interviews with providers allowed them to describe their views and experiences in detail, raise issues that were important to them (e.g., trust building) and explain the rationale behind how the service was being implemented (e.g., the need to be child-led). A strength of our study was that all the providers participated throughout the evaluation, allowing their views and experiences of the service to be explored in real time as it was implemented. This was particularly important because implementation was slow, going through the phases of building trust and intervention development, to finally establishing activities and a referral pathway.

Only six CYP were interviewed, so insight into their views and experiences of the service is limited. We had also aimed to interview CYP referred for additional therapeutic support, but no referral offers were taken up by the CYP during the time of the study so this could not be done.

The fact that the service developed iteratively during our evaluation meant we identified barriers and facilitators to implementation that related to the characteristics of setting in which the service was delivered, and the relationships and exchanges that were needed for the service to be implemented. Our definition of implementation, therefore, was to what extent the service was being operationalised and not to what extent the service was being delivered as intended. This latter definition is the one that is often used when evaluating complex health interventions and is the definition used within the RE-AIM framework developed to guide this process ^28^. This definition, however, assumes the intervention has been fully developed and is clearly defined and structured before being implemented, and that we have some measure or expectation against which to assess implementation. Thus, our paper raises questions about how implementation should be defined and suggests this should depend on to what extent the intervention has been defined prior to it being used in a real-world setting.

## Conclusion

Community settings offer valuable spaces for mental health services for minoritised CYP, ensuring easy access to trusted adults. Successful implementation of youth wellbeing services in community settings require understanding of the context, commitment to relationship building, and flexibility in delivery and pace of the intervention. Future development and evaluation of similar services should acknowledge these requirements.

## Ethical approval

Ethical approval was granted by the University of Bristol Faculty of Health Sciences Research Ethics Committee on 30 ^th^ Nov 2022 (application ID13984).

## Consent to participate

The researcher secured written informed consent in advance and conducted all the interviews. Consent was obtained from the guardians or parents for children under 16 years and from CYP themselves if they were 16 years or older. Consent forms available in supplementary files.

## Data Availability

**Please contact Professor Deborah Caldwell to request for data.** **Because of the very small sample and risk of identification, transcripts will not be made available alongside the manuscript. As very few providers were interviewed, and they were concerned about confidentiality and being identified, we did not ask them to consent to their data being stored and made available for future use. However, parents did consent to their children's data being anonymised, stored and made available to other researchers. These data are therefore available upon request from the lead author, for the purposes of research.** Open Science Framework: Improving wellbeing and mental health support for young people from black, Asian and minoritised groups DOI 10.17605/OSF.IO/X28MR ^22^ This project contains the following extended data: COREQ checklist Supplementary data files Open Science Framework: COREQ Checklist ‘Improving wellbeing and mental health support for young people from black, Asian and minoritised groups’ DOI 10.17605/OSF.IO/X28MR ^22^ Data are available under the terms of the Creative Commons Attribution 4.0 International license (CC-BY 4.0) ( https://creativecommons.org/licenses/by/4.0/).
