# Supplementary material for: Barriers and facilitators to implementation of a community wellbeing service for black, Asian and minoritised young people
Source: NIHR Open Res. 2025 Sep 23;5:46. Originally published 2025 May 15. [Version 2] doi: 10.3310/nihropenres.13912.2 (PMC12800594; doi:10.3310/nihropenres.13912.2)
Supplement: Peer review history

# Reviewer report: Reviewer response for version 2

**DOI:** 10.3310/nihropenres.15356.r38361

**Published:** 2026-01-13

**Authors:** Min K Chong

**Affiliations:** 1 The University of Sydney, Sydney, New South Wales, Australia

This paper evaluates the implementation of a new mental wellbeing service for young people from minoritised ethnic backgrounds delivered within an urban youth centre in the UK. Using a mixed-methods implementation evaluation over the first 12 months, the study draws on longitudinal qualitative interviews with four service practitioners, paired interviews with six young people, fieldnotes, and descriptive attendance data. Thematic analysis of collected data demonstrated that the implementation was facilitated by trust building between practitioners and child and young people, as well as between organisations, while barriers included the informal nature of activities, slow implementation, inconsistent attendance, and young people’s reluctance to engage.

 This is a clearly written and concise paper that addresses an important gap in the implementation literature relating to mental wellbeing services for minoritised young people in community settings. The focus on real-world service implementation, rather than intervention efficacy alone, is a notable strength and aligns well with current priorities in youth mental health research and practice.

 Overall, the paper demonstrates a novel and timely approach to embedding mental wellbeing support within community settings for marginalised young people, particularly those who may have limited access to and information about mental health services. The emphasis on flexibility, relationship building, and iterative service development offers valuable insights for future community-based service design. Further clarification on aspects of the service structure, analytic approach, and interpretation of findings would further strengthen the paper.

**Introduction**

- The supplementary file referenced in the paper was not available for review, therefore was not able to review the guided questions.
- The paper describes an iterative process of developing service aims and activities, noting that *“the aims of the new service were broad, so the specific design and professional activities of the service were not decided a priori and were developed as the service was implemented.”*
  However, further details on how the four components of the service were developed and why these components were deemed suitable for this demographic were not provided. Please consider adding this to the results or supplementary material.
- It would be helpful to specify explicitly that the two-hour weekly sessions were group-based activities

**Methods **

- The longitudinal interview design and inclusion of all service practitioners are key strengths of the study. In particular, the longitudinal format allows insight into whether practitioners’ perceived facilitators and barriers evolved over time. It would be useful to:
-
  - Identify the timepoints at which participant quotes were collected.
  - Clarify whether practitioners’ views on service design or implementation strategies changed across interviews.
- The interviewer’s existing relationship with interviewees may have limited the transparency of the data collected. It may be worth acknowledging that participants could have been hesitant to provide critical feedback due to their existing relationship with the researcher
- The following description of data collection and analysis appears duplicative and potentially counterintuitive:

*“In terms of analysing the interview data, following the principles of grounded theory, data collection and analysis proceeded in parallel… The interview transcripts were analysed thematically, using the approach suggested by Braun and Clarke.”*

 As thematic analysis also does not prescribe a fixed order of data collection and analysis, a clearer justification of the use of both grounded theory principles and thematic analysis would strengthen methodological clarity.

- Providing information on theme development over time (e.g. a thematic map or description of analytic iteration) would further demonstrate reflexivity.

**Results**

- Leaving the service design broad is a strength, as co-development and iterative refinement can better meet young people’s needs. However, as previously stated, further explanation of how and why the four service components evolved (or remained the same) over time would be beneficial
- The themes *“Fostering trust and building relationships”* and *“Trust building between the organisations”* appear conceptually similar. A clearer distinction between trust-building with child and young people versus trust-building between organisations would improve interpretability.
- Minor clarification: in the quote *“So will show up for a week, two weeks,”* it is unclear whether “So” was intended to be “Some.”

**Discussion**

- The opening sentence of the discussion states that implementation was facilitated by practitioners’ lived experience and iterative, tailored service development. However, the data presented do not clearly demonstrate how lived experience or iteration directly influenced implementation, and this claim may require further support or reframing.
- The 5 young people referred for further care did not take the offer. This may provide insight into the challenges or limitations of this service design. Further exploration on this may be important.

Is the work clearly and accurately presented and does it cite the current literature?

Yes

If applicable, is the statistical analysis and its interpretation appropriate?

Not applicable

Are all the source data underlying the results available to ensure full reproducibility?

Yes

Is the study design appropriate and is the work technically sound?

Yes

Are the conclusions drawn adequately supported by the results?

Partly

Are sufficient details of methods and analysis provided to allow replication by others?

Yes

Reviewer Expertise:

Youth mental health, health services research

I confirm that I have read this submission and believe that I have an appropriate level of expertise to confirm that it is of an acceptable scientific standard, however I have significant reservations, as outlined above.

# Reviewer report: Reviewer response for version 2

**DOI:** 10.3310/nihropenres.15356.r37294

**Published:** 2025-10-08

**Authors:** Maria Loades, Sophie Dallison

**Affiliations:** 1 University of Bath Department of Psychology (Ringgold ID: 228664), Bath, England, UK; 2 Psychology, University of Bath (Ringgold ID: 1555), Bath, England, UK

The authors have sufficiently addressed the initial feedback given. No further comments to make.

Is the work clearly and accurately presented and does it cite the current literature?

Partly

If applicable, is the statistical analysis and its interpretation appropriate?

Not applicable

Are all the source data underlying the results available to ensure full reproducibility?

No source data required

Is the study design appropriate and is the work technically sound?

Partly

Are the conclusions drawn adequately supported by the results?

Yes

Are sufficient details of methods and analysis provided to allow replication by others?

Partly

Reviewer Expertise:

Child and adolescent mental health provision; clinical psychology

We confirm that we have read this submission and believe that we have an appropriate level of expertise to confirm that it is of an acceptable scientific standard.

# Reviewer report: Reviewer response for version 1

**DOI:** 10.3310/nihropenres.15120.r35920

**Published:** 2025-07-21

**Authors:** Gerard Leavey

**Affiliations:** 1 Ulster University, Coleraine, Northern Ireland, UK

The authors indicate that young people from black and minority ethnic backgrounds are underrepresented in CAMHS services, despite evidence that they may be more vulnerable to mental health problems than their white counterparts. They mention the need for more culturally appropriate services.

 The report provides information related to the evaluation of a  new "wellbeing service" for minoritized young people in an urban youth center. There are some issues related to the impact of the service that are covered here but they are only sketchily presented. 

 Although they have set up this new wellbeing service, there is no mention of the specific design, aims and professional activities of the service.  (a) For whom specifically was the service intended and what processes were set up - was it a universal programmed or targeted at CYP thought to be at risk? (b) I note that it wasn't codeveloped with the CYP but rather the service providers - was this a problem or potentially a failure; (c) How many CYP used the service overall and how many were approached for the study; (d) what was the ethnic make-up of the youth center and the study participants - do the authors feel this is relevant, and if not, why not? and related (e) where does cultural appropriateness fit in this programmed?

 The conclusions  in the abstract are somewhat banal (requirements for trust and flexibility) whereas the findings in the lay summary provided much more useful information. On these points - it would be useful to see the challenges elaborated more fully. For example, that CYP don't necessarily regard themselves as having a mental health problem and were wary of the 'wellbeing' label for the service. Again, sensitivity to stigma and terminology which may have emerged in a better co-design. This could be covered better. 

 The report could provide more details on the topic guide and the analysis.

Is the work clearly and accurately presented and does it cite the current literature?

Partly

If applicable, is the statistical analysis and its interpretation appropriate?

Not applicable

Are all the source data underlying the results available to ensure full reproducibility?

Partly

Is the study design appropriate and is the work technically sound?

Partly

Are the conclusions drawn adequately supported by the results?

Yes

Are sufficient details of methods and analysis provided to allow replication by others?

Partly

Reviewer Expertise:

mental illness, ethnicity and culture, Health services research, qualitative design

I confirm that I have read this submission and believe that I have an appropriate level of expertise to confirm that it is of an acceptable scientific standard, however I have significant reservations, as outlined above.

# Response

**Published:** 2025-09-19

**Authors:** Sharea Ijaz

**Affiliations:** PHS, University of Bristol, Bristol, England, UK

Thank you for your comments that have helped us improve the clarity of our paper. We have addressed the comments by adding details in the manuscript for the points raised.

- The aims of the service, its design and activities were broad and developed as service was implemented. They key aims were to support attending minoritised young people’s mental wellbeing and facilitate appropriate referrals to mental health services. We have now added in the following text in the introduction section to elaborate on the aims and design process for the service as well as target population: 

*A new community-based mental wellbeing service was commissioned by Bristol’s local authority to improve wellbeing support and access to mental health services for CYP from Black and Asian minority ethnicities. To set up the service, the local authority organised a meeting with Black and Asian led CYP support organisations in the city to identify those willing to collaborate on service delivery in a community setting. The organisations in one locality who agreed were contracted to collaborate for developing and providing the service and supporting an evaluation. The key service aims were to support minoritised young people’s mental wellbeing and facilitate appropriate referrals to mental health services. The service was set in a youth club and intended to be both universal (through games and activities for all attendees) and targeted (through mentoring and/ or referral for at risk CYP). *

- We have clarified on how many CYP attended the new service and were approached for interview. The text now reads: 

*94 CYP attended at least one wellbeing session during the course of the study. All of them were shown study flyers and leaflets and approached for participation in the study.*

- We have added text describing ethnic makeup of the youth centre and the cultural context of the service providers who developed the service content as follows:

*The ethnic makeup of the youth centre attendees was varied, including CYP with Black, Asian and mixed heritages in majority.*

*The service hub was a 2-hour, weekly, youth activity session run for 11- to 17-year-old CYP in the youth centre where mental health practitioners were present. The aims of the new service were broad, so the specific design and professional activities of the service were not decided a priori and were developed as service was implemented. It was co-developed and facilitated by two youth practitioners from the community centre and two specialist mental health practitioners from the mental health organisation (the service ‘providers’). Majority of the youth and mental health practitioners were from the same community and of Black Asian or Mixed ethnicity. The practitioners discussed and agreed on what the service would comprise of and developed it together, based on their knowledge and experience of looking after minoritised CYPs and their own lived experience of being from minoritised communities. They therefore understood the reluctance and sensitivity around mental health.*

*We held in person and online conversations prior to and during the study with parents and guardians whose children attended the youth centre to inform them about the study. These conversations indicated that parents trusted the setting providers for looking after their children because of their longstanding presence in the community and shared values.*

- We have added the challenges to the abstract conclusions. The text now reads: 

*Successful implementation of wellbeing services in community settings for minoritised young people can be affected by the informal and relaxed nature of the setting and the activities delivered, and the extent to which young people are willing to engage.  Additionally, it requires relationship building and flexibility in delivery and pace. Future development and evaluation of similar services should consider these requirements.*

- We have added the following text to elaborate on our qualitative analysis methods and report the topic guides in full in supplementary files:

*In terms of analysing the interview data, following the principles of grounded theory[23], data collection and analysis proceeded in parallel, so that findings from earlier interviews informed the focus of later data collection. The interview transcripts were  analysed thematically, using the approach suggested by Braun and Clarke [24]. Data collection and analysis proceeded in parallel, so that findings from earlier interviews informed the focus of later data collection. This entailed a subsample of all transcripts being independently read and manually coded by SS and KT, who then met to discuss their coding and interpretation of the data, and to develop a coding frame which reflected themes identified in the data. Once the coding frame had been agreed, transcripts were uploaded into NVivo v14 [23] and electronically coded. As data collection progressed, new codes were added to the coding frame and transcripts that had previously been coded were re-coded where necessary. Finally, codes were grouped together to develop categories and collated into potential themes and sub-categories.* *This inductive approach to the analysis ensured findings were based on participants’ accounts and not coded according to the researchers’ pre-existing assumptions or priorities. Researcher bias was also controlled for through the double coding of transcripts and regular discussion between SS and KT about how findings related to the data set. In addition, during data collection, SS discussed interim findings with providers to check our interpretation of the data. Whilst it was felt SS’s ethnicity had facilitated access to the centre and the interviews with providers, it was also felt her gender had hindered the extent to which young men, particularly the 16- and 17-year-olds, using the centre were willing to take part in the research.*

# Reviewer report: Reviewer response for version 1

**DOI:** 10.3310/nihropenres.15120.r35819

**Published:** 2025-06-17

**Authors:** Maria Loades, Sophie Dallison

**Affiliations:** 1 Psychology, University of Bath (Ringgold ID: 1555), Bath, England, UK; 2 University of Bath Department of Psychology (Ringgold ID: 228664), Bath, England, UK

This article presents a valuable description of the implementation of a novel and innovative service designed specifically for minoritised young people. The authors have commendably embedded patient and public involvement throughout the research process and drawn on a rich variety of data sources—including field notes, interviews with both young people and service providers, and basic count data on service attendance. As such, this is a mixed methods study, though the data is primarily qualitative in nature.

 Overall, the paper makes a useful contribution to the existing literature, particularly by addressing an important gap in understanding how services can be better tailored to meet the needs of young people from minoritised backgrounds.

 That said, there are a few areas where the manuscript could be further strengthened. Most of these are relatively minor, but three stand out as more substantive.

 First, some content currently located in the results section might be better placed in the methods section. Moving this information would help readers more clearly understand the study context before engaging with the findings. In particular, restructuring the methods section using conventional subheadings—such as Participants, Procedure, Materials and Measures, Ethical Approval, and Data Analysis—could improve clarity and flow. For example, the description of participants that appears at the beginning of the results section could be moved to the newly created Participants subsection. While approaches can vary, presenting this information earlier would reduce repetition and better support the reader’s journey through the manuscript, reserving the results section for presentation of findings.

 Second, the description of the data analysis process is currently quite brief. Given the use of an inductive thematic analysis, we encourage the authors to provide a more detailed account of how the analysis was conducted. It would be helpful to reference the specific approach followed—for instance, whether it aligns with Braun and Clarke’s thematic analysis—and to describe the epistemological and ontological positioning that underpinned the analysis. A brief comment on reflexivity in relation to the analytic process would also strengthen the manuscript by helping readers contextualise the findings, or at a minimum a statement on each researchers’ positionality.

 Third, it doesn’t truly seem to be a mixed methods study as the quantitative data are very limited, please consider whether it is appropriate to call it such.

 In addition to these main points, we would also encourage the authors to consider the following more minor suggestions:

1. The plain English summary is still quite complex and would be hard to follow in places for a lay reader – we encourage the authors to review and simplify where possible. The NIHR has useful guidance around lay summaries.
2. It may be helpful to include the topic guides used for interviews as supplementary material, to support transparency and replicability.
3. While the importance of preserving participant anonymity is fully appreciated, including some basic demographic data—for instance, the mean or median age of participants alongside the age range (which we understand to be 11–15)—would provide valuable context without compromising confidentiality. Similarly, a summary indication of whether participants shared the same minoritised background or represented a range of minoritised groups would help readers consider the transferability of findings to other settings.
4. A brief definition of what a young people’s advisory group is needed for reader who are not familiar with these.
5. Results: it was a valuable inclusion to have a paragraph outlining the *barriers* before delving into each theme, however this was missing for the *facilitators* section, so it would be helpful to have a paragraph summarising the themes and for consistency.
6. Finally, it could strengthen the paper if the authors briefly referenced a relevant implementation framework—such as CFIR or RE-AIM—in either the introduction or, more pertinently, the discussion. This framing does not need to be extensive but could support interpretation of the findings and help to situate them within broader implementation science literature. It may also assist in further identifying and framing the directions for future research. In keeping with implementation, it is unclear what ‘implemented’ means here: “The new service was **implemented** during a 2-hour, weekly, youth activity session ….”, perhaps a different word would better explain this.

There are a couple of typos and grammatical errors so a thorough proofread could address these:

- Consistency needed on US/UK spelling e.g. minoritised/minoritized
- Check for full stops and missing words
- Use of the phrase ‘de novo’ – unclear what this means/jargon
- Use of ‘(see below)’ is unclear where the authors are directing the reader

Thank you for the opportunity to engage with this important and timely work.

Is the work clearly and accurately presented and does it cite the current literature?

Partly

If applicable, is the statistical analysis and its interpretation appropriate?

Not applicable

Are all the source data underlying the results available to ensure full reproducibility?

No source data required

Is the study design appropriate and is the work technically sound?

Partly

Are the conclusions drawn adequately supported by the results?

Yes

Are sufficient details of methods and analysis provided to allow replication by others?

Partly

Reviewer Expertise:

Child and adolescent mental health provision; clinical psychology

We confirm that we have read this submission and believe that we have an appropriate level of expertise to confirm that it is of an acceptable scientific standard, however we have significant reservations, as outlined above.

# Response

**Published:** 2025-09-19

**Authors:** Sharea Ijaz

**Affiliations:** PHS, University of Bristol, Bristol, England, UK

Thank you for your feedback and suggestions which have helped improve the clarity of the paper.

 For the main points raised:

1. We have moved the details of participants to methods section and added headings for different parts of the methods as suggested.
2. We have added more details on analysis as suggested along with references to underpinning frameworks and comments on reflexivity in the analysis process. The text now reads: *In terms of analysing the interview data, following the principles of grounded theory* *[23]* *,* *data collection and analysis proceeded in parallel, so that findings from earlier interviews informed the focus of later data collection. The interview transcripts were  analysed thematically, using the approach suggested by Braun and Clarke* *[24]* *.* *This inductive approach to the analysis ensured findings were based on participants’ accounts and not coded according to the researchers’ pre-existing assumptions or priorities.  Researcher bias was also controlled for through the double coding of transcripts and regular discussion between SS and KT about how findings related to the data set.  In addition, during data collection, SS discussed interim findings with providers to check our interpretation of the data. Whilst it was felt SS’s ethnicity had facilitated access to the centre and the interviews with providers, it was also felt her gender had hindered the extent to which young men, particularly the 16- and 17-year-olds, using the centre were willing to take part in the research.* 
3. We have revised the title to remove reference to a mixed method design.

For the minor points we have addressed these as follows:

1. We have revised the text of the plain language summary to make it simpler and can confirm the readability age to be at 8 ^th^ grade now according to Flesch-Kincaid Grade Level.
2. All topic guides are available in supplementary files on Open Science Framework as per NIHR Open guidance: OSF | Improving wellbeing and mental health support for young people from black, Asian and minoritized groups and have been linked as a reference in the manuscript text: *(see Supplementary data for topic guides* *[22]). *
3. We have added the median age, and a reference to the range of ethnic background and for participants as advised on page 10. The text now reads: *The six young people interviewed were between 11 and 15 years (Median 13 years). The young people included both boys and girls and represented a range of minoritized ethnic backgrounds.*
4. We have added a definition of YPAG as suggested: *A YPAG is a group of volunteer young people who advise on research concerning young people using their lived experience and age specific views.* 
5. We have added an introduction sentence to facilitators as suggested: *Facilitators to implementation included building trust and relationships with service users and between organisations, and tailoring activities to context.*
6. We appreciate this comment as it has led to us to think about how we were defining implementation during the study. We have now clarified this in the paper, within the discussion, by inserting the following text: *The fact that the service developed iteratively during our evaluation meant we identified barriers and facilitators to implementation that related to the characteristics of setting in which the service was delivered, and the relationships and exchanges that were needed for the service to be implemented. Our definition of implementation, therefore, was to what extent the service was being operationalised and not to what extent the service was being delivered as intended. This latter definition is the one that is often used when evaluating complex health interventions and is the definition used within the RE-AIM framework developed to guide this process [28].  This definition, however, assumes the intervention has been fully developed and is clearly defined and structured before being implemented, and that we have some measure or expectation against which to assess implementation.  Thus, our paper raises questions about how implementation should be defined and suggests this should depend on to what extent the intervention has been defined prior to it being used in a real-world setting. *  

We have also done a thorough check and addressed the grammatical, jargon and proofing errors *.*
